# Prediction of MHC class II binding affinity using SMM-align, a novel stabilization matrix alignment method

**DOI:** 10.1186/1471-2105-8-238

**Published:** 2007-07-04

**Authors:** Morten Nielsen, Claus Lundegaard, Ole Lund

**Affiliations:** 1 Center for Biological Sequence Analysis, BioCentrum-DTU, Technical University of Denmark, Lyngby, Denmark

## Abstract

**Background:**

Antigen presenting cells (APCs) sample the extra cellular space and present peptides from here to T helper cells, which can be activated if the peptides are of foreign origin. The peptides are presented on the surface of the cells in complex with major histocompatibility class II (MHC II) molecules. Identification of peptides that bind MHC II molecules is thus a key step in rational vaccine design and developing methods for accurate prediction of the peptide:MHC interactions play a central role in epitope discovery. The MHC class II binding groove is open at both ends making the correct alignment of a peptide in the binding groove a crucial part of identifying the core of an MHC class II binding motif. Here, we present a novel stabilization matrix alignment method, SMM-align, that allows for direct prediction of peptide:MHC binding affinities. The predictive performance of the method is validated on a large MHC class II benchmark data set covering 14 HLA-DR (human MHC) and three mouse H2-IA alleles.

**Results:**

The predictive performance of the SMM-align method was demonstrated to be superior to that of the Gibbs sampler, TEPITOPE, SVRMHC, and MHCpred methods. Cross validation between peptide data set obtained from different sources demonstrated that direct incorporation of peptide length potentially results in over-fitting of the binding prediction method. Focusing on amino terminal peptide flanking residues (PFR), we demonstrate a consistent gain in predictive performance by favoring binding registers with a minimum PFR length of two amino acids. Visualizing the binding motif as obtained by the SMM-align and TEPITOPE methods highlights a series of fundamental discrepancies between the two predicted motifs. For the DRB1*1302 allele for instance, the TEPITOPE method favors basic amino acids at most anchor positions, whereas the SMM-align method identifies a preference for hydrophobic or neutral amino acids at the anchors.

**Conclusion:**

The SMM-align method was shown to outperform other state of the art MHC class II prediction methods. The method predicts quantitative peptide:MHC binding affinity values, making it ideally suited for rational epitope discovery. The method has been trained and evaluated on the, to our knowledge, largest benchmark data set publicly available and covers the nine HLA-DR supertypes suggested as well as three mouse H2-IA allele. Both the peptide benchmark data set, and SMM-align prediction method (*NetMHCII*) are made publicly available.

## Background

Major histocompatibility complex molecules (MHCs) play an essential role in the host pathogen interactions determining the onset of a host immune response. One arm of the cellular immune system is guided by the MHC class I complexes that present peptides derived from intra cellular proteins to cytotoxic T cell circulating in the blood periphery. The MHC class II complexes guide the other arm of the cellular immune system. These complexes present peptides derived from endocytosed proteins to CD4+ helper T lymphocytes (HTLs) to stimulate cellular and humoral immunity against the pathogenic microorganism.

Predicting the peptides that bind to MHC class II molecules can effectively reduce the number of experiments required for identifying helper T cell epitopes and play an important role in rational vaccine design. Large efforts have been invested in deriving such prediction methods. In general terms, the different methods can be classified in two groups. One group being quantitative matrices estimated from experimentally derived position specific binding profiles [1-3], and the other group comprising data driven bioinformatical motif search methods. The number of different bioinformatical methods proposed to predict MHC class II binding is large and growing including Gibbs samplers [4], Ant colony [5], Artificial neural networks [6], Support vector machines [7,8], hidden Markov models [9], and other motif search algorithms [10-12]. However, most of these methods have been trained and evaluated on very limited data sets covering only a single or a few different MHC class II alleles. Further the majority of the methods are trained on MHC ligand data (peptides eluted from MHC complexes present on the cell surface). This type of qualitative prediction methods are well suited to classify data in to binders and non-binders, but they do not allow a direct prediction of the peptide:MHC binding strength.

Recently, a large set of quantitative MHC class II peptide-binding data has been made publicly available on the IEDB databases [13]. The data set comprises peptide data with IC50 binding affinities for more than 14 HLA (human MHC) and several mouse MHC class II alleles.

Here, we present a novel method, SMM-align, for quantitative MHC class II binding predictions. The method is an extension of the stabilization matrix method proposed by Peters et al. [14]. The SMM-align method seeks to identify a weight matrix that optimally reproduces the measured IC50 values for each peptide in the training set.

The method allows for identification the MHC class II binding motif in terms of a position specific weight matrix. The output of the SMM-align method is IC50 binding affinity values, enabling direct readout of the peptide:MHC binding affinity.

To our knowledge, only three other methods are publicly available for quantitative MHC class II prediction, namely the ARB [12], SVRMHC [7] and MHCpred [15] methods. Other methods such as SVMHC [16] and Propred [17] are implementations of the TEPITOPE method [3], and provide prediction scores that are not in any direct way proportional to the peptide binding affinity. Both the SVRMHC and MHCpred methods are trained on relatively small sets of quantitative peptide binding data contained within the AntiJen database [18] and could probably improve if retrained on the data used here. The SVRMHC method covers six, and MHCpred three MHC class II alleles, respectively. The ARB method is trained on quantitative peptide binding data available within the IEDB database [13]. The TEPITOPE is an experimentally derived virtual matrix based prediction method that covers 50 different HLA-DR alleles, and relies on the approximation that the peptide binding specificity can be determined solely from alignment of MHC pockets amino acids [3].

In this work, we design a large-scale benchmark calculation covering 14 HLA-DR and three mouse H2-IA alleles. We compare the predictive performance of five methods in terms of their ability to predict binding affinity of more than 4600 peptides. The methods included in the benchmark are; SMM-align, Gibbs sampler [4], SVRMHC [7], MHCpred [15], ARB [12] and TEPITOPE [3].

## Results

The SMM-align method was applied to derive a position specific scoring matrix for prediction of MHC-II binding affinities for each of the 14 HLA-DR and three H2-IA alleles in the benchmark dataset. The predictive performance of the method was estimated using five-fold cross-validation.

### Cross-validated predictive performance

The predictive performance of the SMM-align method is compared to that of the Gibbs sampler, TEPITOPE, SVRMHC, MHCpred, and ARB methods. The SMM-align, Gibbs sampler, and ARB methods cover all 14 alleles. TEPITOPE covers 11, SVRMHC five, and MHCpred only three of the 14 alleles. The predictive performance of the different methods was measured in terms of the area under the ROC curve (AUC) [19], the Pearson correlation [20], and the Spearman's rank correlation [20]. Since the TEPITOPE method does not produce prediction values that are linearly related to the log-transformed IC50 binding affinities, the use of the Pearson correlation coefficient would be an inappropriate measure for the prediction accuracy for this method. Hence, we for this method only evaluate the predictive performance using the other two measures.

Table 1 gives a summary of the HLA-DR benchmark calculation results.

**Table 1:** Summary of the HLA-DR benchmark results.

**AUC**									
**Allele**	**SMM**	**Gibbs**	**TEPITOPE**	**SVRMHC**	**MHCpred**	**ARB**	**SMM-PRF**	**NetMHCII**	**N**

**A**	0.730	0.697				0.758	0.749	0.756	14
**B**	0.740	0.705	0.736			0.762	0.748	0.750	11
**C**	0.710	0.690	0.714	0.688		0.719	0.719	0.722	5
**D**	0.737	0.710	0.723		0.606	0.717	0.749	0.754	3

**Pearson correlation**									

**Allele**	**SMM**	**Gibbs**	**TEPITOPE**	**SVRMHC**	**MHCpred**	**ARB**	**SMM-PRF**	**NetMHCII**	**N**

**A**	0.420	0.368				0.464	0.436	0.448	14
**C**	0.408	0.369		0.157		0.431	0.428	0.435	5
**D**	0.458	0.384			0.218	0.425	0.480	0.487	3

**Spearman's rank correlation**									

**Allele**	**SMM**	**Gibbs**	**TEPITOPE**	**SVRHMM**	**MHCpred**	**ARB**	**SMM-PRF**	**NetMHCII**	**N**

**A**	0.430	0.372				0.464	0.445	0.453	14
**B**	0.443	0.378	0.428			0.479	0.458	0.463	11
**C**	0.398	0.353	0.430	0.377		0.424	0.422	0.427	5
**D**	0.450	0.365	0.434		0.210	0.407	0.474	0.481	3

The predictive performance is shown in terms of the average area under the ROC curve (upper panel), the average Pearson's correlation (middle panel), and the average Spearman's rank correlation (lower panel) for the SMM (SMM-align), Gibbs sampler [4], TEPITOPE [3], SVRMHC [7], MHCpred [15], and ARB [12] methods, respectively. The SMM-PRF method refers to the extended SMM align method including penalties for longer peptides and short amino terminal peptide flanking residues, and the *NetMHCII *method refers to the extended SMM align method including direct encoding of peptide flanking residues and penalties for longer peptides and short amino terminal peptide flanking residues. The last column gives the number of alleles included in each average. In A is shown the average performance for all 14 HLA-DR alleles, in B the average performance for the subset of 11 alleles covered by the TEPITOPE method, in C the average performance for the five alleles covered by the SVRMHC method, and in D the average performance for the three alleles covered by the MHCpred method. For each allele, the performance of the SMM-align, Gibbs sampler, and *NetMHCII *methods was estimated using five-fold cross-validation as described in Methods.

The table demonstrates the predictive power of the SMM-align method as compared to that of the other methods. Note that caution should be taking when evaluating the predictive performance of the ARB method. This method has been trained on data from the IEDB database, and thus very likely has been trained on data included in the benchmark evaluation set. The Gibbs sampler has relative poor performance compared to the SMM-align method. There are many possible reasons for this low performance. Most importantly, the Gibbs sampler method is trained on qualitative data only. Before applying the Gibbs sampler method, the data are classified into binders and non-binders, and only the set of binders are included when estimating the binding motif weight matrix. The SMM-align method, on the other hand, includes both binding and non-binding data when estimating the motif weight matrix.

The details of the benchmark calculation as evaluated in terms of the AUC performance measure are shown in Table 2 (evaluation in terms of the Pearson's and Spearman's rank correlations are shown in Supplementary materials table 1 [see Additional file 1]).

**Table 2:** Details of the benchmark calculation covering the 14 HLA-DR alleles.

**AUC**									
**Allele**	**SMM**	**Gibbs**	**TEPITOPE**	**SVRMHC**	**MHCpred**	**ARB**	**SMM -PRF**	**NetMHCII**	**N**

1*0101	0.702	0.676	0.647	0.623	0.565	0.666	0.716	0.716	1203
1*0301	0.779	0.722	0.734			0.799	0.770	0.765	474
1*0401	0.741	0.759	0.754	0.739	0.606	0.737	0.756	0.758	457
1*0404	0.798	0.743	0.829			0.788	0.808	0.785	168
1*0405	0.727	0.724	0.790	0.701		0.724	0.733	0.735	171
1*0701	0.768	0.695	0.768		0.647	0.749	0.774	0.787	310
1*0802	0.724	0.721	0.769			0.803	0.740	0.756	174
1*0901	0.726	0.734				0.711	0.759	0.775	117
1*1101	0.715	0.715	0.710			0.727	0.720	0.734	359
1*1302	0.810	0.716	0.720			0.917	0.819	0.818	179
1*1501	0.715	0.672	0.726	0.730		0.792	0.733	0.736	365
3*0101	0.620	0.512				0.717	0.771	0.815	102
4*0101	0.730	0.742				0.800	0.729	0.736	181
5*0101	0.664	0.618	0.653	0.649		0.677	0.655	0.664	343

The predictive performance is shown in terms of the area under the ROC curve (AUC) for the SMM-align, Gibbs sampler [4], TEPITOPE [3], SVRMHC [7], MHCpred [15], and ARB methods, respectively. The SMM-PRF method refers to the extended SMM align method including penalties for long peptides and short amino terminal peptide flanking residues, and the *NetMHCII *method refers to the final extended SMM align method including direct encoding of peptide flanking residues and penalties for longer peptides and short amino terminal peptide flanking residues. The first column gives the allele names as 1*0101 for DRB1*0101 etc The last column gives the number of peptide data included for each allele. For each allele, the performance of the SMM-align, Gibbs sampler, and *NetMHCII *methods was estimated using five-fold cross-validation as described in the text. The details of the benchmark calculation as measured in terms of the Pearson's and Spearman's rank correlation are shown in Supplementary materials table 1 [see Additional file 1].

The SMM-align, ARB and TEPITOPE methods all have similar predictive performances. Comparing to the other methods the SMM-align method has the highest predictive performance, followed by the Gibbs sampler and SVRMHC methods. The MHCpred method performs the poorest. The direct relation between the SMM-align prediction scores and the log-transformed IC50 binding affinity is confirmed by fact that a least square linear fit to the log-transformed binding data as a function of the SMM-align prediction values has a slope close to unity (data not shown).

### Incorporating the peptide length in prediction of peptide:MHC class II binding

Chang et al. [11] recently proposed a strategy for incorporating of peptide length into prediction of peptide-MHC class II binding, and demonstrated that at least for some alleles the approach lead to significant improvements in prediction accuracy.

Here, we evaluate the peptide length-based approach on two data sets covering the three alleles included in the work by Chang et al. [11]. The data are taken from two sources. AntiJen: Data from the AntiJen database as downloaded from the supplementary data in Chang et al., and IEDB: Data from the IEDB database [13]. For each allele in the two data sets, we train the SMM-align method in three distinct manners; a) using no peptide length information, b) including a peptide length affinity baseline estimated from the training data as described by Chang et al., and c) including a peptide length affinity baseline estimated from the data in the alternative data set, i.e., using the DRB1*0101 AntiJen data set to estimate the baseline correction for the training of the IEDB DRB1*0101 data and vise versa. The result of the benchmark calculation is shown in Table 3.

**Table 3:** Predictive performance in terms of the area under the ROC curve (AUC) of the different methods evaluated on six data sets.

	**AntiJen**			**IEDB**		
	
	DRB1*0101	DRB1*0401	DRB1*1501	DRB1*0101	DRB1*0401	DRB1*1501
**ISC-PLC**	0.709	0.757	0.609			
**SMM-align**	0.718	0.806	0.691	0.702	0.741	0.715
**TEPITOPE**	0.667	0.744	0.665	0.647	0.754	0.726
**Chang**	0.770	0.757	0.677			
**SMM-regr**	0.807	0.819	0.741	0.744	0.750	0.718
**SMM-regr-alter**	0.616	0.785	0.669	0.645	0.721	0.712
**SMM-PFR**	0.742	0.814	0.726	0.716	0.756	0.733

The methods are; ISC-PLS [15], SMM-align, TEPITOPE, Chang [11], SMM-regr (SMM with peptide length regression correction from training data set), SMM-regr-alter (SMM with peptide length regression correction from alternative AntiJen/IEDB dataset), and SMM-PFR (The SMM-PRF method refers to the extended SMM align method including penalties for long peptides and short amino terminal peptide flanking residues). The data sets consist of peptides binding data from two sources (IEDB and AntiJen) covering three HLA-DR alleles (1*0101, 1*0401, and 1*1501). Performance value for the ISC-PLS and Chang methods, are taken from Chang et al. [11]. These values are only available for the AntiJen data set.

Focusing on the first three rows in the table comparing, the predictive performance of the SMM method is seen to compare favorably to that of both ISC-PLS, and TEPITOPE. The average predictive performance in terms of AUC for the ISC-PLC, TEPITOPE, and SMM methods on the three alleles in the AntiJen data sets is 0.692, 0.692, and 0.738, respectively. Further, the table shows that the performance gain proposed the Chang et al. for the AntiJen DRB1*0101, and DRB1*1501 allele data sets is recovered in our implementation (see SMM-regr). However, it is striking to observe that the alternative baseline correction consistently for all alleles leads to a dramatic drop in predictive performance (see SMM-regr-alter). This suggests that the baseline subtraction, rather than being alleles specific, is highly data set dependent and that the performance gain observed including an affinity baseline correction does not necessarily reflect a genuine feature of MHC class II binding predictions, but may arise as a result of over-fitting the method to a length distribution and binding profile of a particular data set.

We observed a large discrepancy between the affinity-length profiles for the peptide data in the AntiJen, IEDB and SYFPEITHI databases (details of the calculation are shown in Supplementary materials, Figure 1 [see Additional file 2]). The short peptides (length < 15 amino acids) in the IEDB data set, seems to follow an affinity profile in agreement with the observed length profile for natural MHC-II ligands in the SYFPEITHI database. This is in contrast to what was observed for the peptides the AntiJen data set. For longer peptides, both the AntiJen and IEDB data sets followed a similar affinity profile that deviated strongly from the length profile of natural MHC-II ligands. The large discrepancy between the affinity-length profiles for the two databases provides a possible explanation as to why the alternative baseline correction gives so poor predictive performance. While the AntiJen data sets tend to have high binding affinities for short peptides, the opposite is the case for the IEDB data sets, and applying an AntiJen derived baseline correction to an IEDB data set thus could give no improvement to the prediction results.

**Figure 1:**
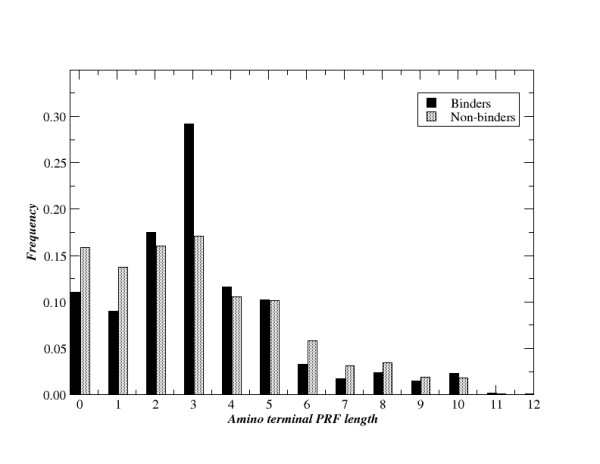
Length distribution of amino terminal PFRs for MHC-II binding and non-binding peptides. All peptide data for the three alleles in the AntiJen and IEDB data sets are included in the figure. Binding peptides have an affinity stronger than 500 nM. The PFR is defined as the residues flanking the peptide-binding core as determined by the SMM-align method.

### Including peptide flanking residues

Looking at differences in the length distribution of the amino terminal peptide flanking residues (PFR) as identified by the SMM alignment method, suggest a feature common to both the AntiJen and IEDB data that separates binding from non-binding peptides.

In Figure 1, the length distribution of the amino terminal PFRs is shown for the combined set of binding and non-binding peptides in the AntiJen, and IEDB data sets, respectively. In the figure, a PFR length of zero indicates that the MHC-II binding core starts at the amino terminal of the peptide leaving a flanking region of zero amino acids. From the figure, it is apparent that a significantly larger fraction of the non-binding peptides have amino terminal PFRs shorter than 2 amino acids (p < 0.001, Fishers exact test), suggesting that amino terminal PFRs do play an important role in stabilizing the peptide:MHC complex. A similar picture is not observed at the C terminal (data not shown).

The requirement for presence of amino terminal flanking amino acids in combination with the observation that the SMM algorithm tends to over-predict the binding affinity for longer peptides (data not shown), suggests a simple scheme suitable for modifying the SMM predictions

S'={S−p, if amino PFR length<2S−p, if peptide length >15S, otherwise
 MathType@MTEF@5@5@+=feaafiart1ev1aaatCvAUfKttLearuWrP9MDH5MBPbIqV92AaeXatLxBI9gBaebbnrfifHhDYfgasaacH8akY=wiFfYdH8Gipec8Eeeu0xXdbba9frFj0=OqFfea0dXdd9vqai=hGuQ8kuc9pgc9s8qqaq=dirpe0xb9q8qiLsFr0=vr0=vr0dc8meaabaqaciaacaGaaeqabaqabeGadaaakeaacqWGtbWucqGGNaWjcqGH9aqpdaGabaqaauaabaqGceaaaeaacqqGtbWucqGHsislcqqGWbaCcqqGSaalcqqGGaaicqqGPbqAcqqGMbGzcqqGGaaicqqGHbqycqqGTbqBcqqGPbqAcqqGUbGBcqqGVbWBcqqGGaaicqqGqbaucqqGgbGrcqqGsbGucqqGGaaicqqGSbaBcqqGLbqzcqqGUbGBcqqGNbWzcqqG0baDcqqGObaAcqGH8aapcqqGYaGmaeaafaqaaeGabaaabaGaee4uamLaeyOeI0IaeeiCaaNaeeilaWIaeeiiaaIaeeyAaKMaeeOzayMaeeiiaaIaeeiCaaNaeeyzauMaeeiCaaNaeeiDaqNaeeyAaKMaeeizaqMaeeyzauMaeeiiaaIaeeiBaWMaeeyzauMaeeOBa4Maee4zaCMaeeiDaqNaeeiAaGMaeeiiaaIaeyOpa4JaeeymaeJaeeynaudabaGaee4uamLaeeilaWIaeeiiaaIaee4Ba8MaeeiDaqNaeeiAaGMaeeyzauMaeeOCaiNaee4DaCNaeeyAaKMaee4CamNaeeyzaugaaaaaaiaawUhaaaaa@7DE3@

where S is the original prediction score from the SMM method, and p is a parameter determining the penalty for short PFRs and longer peptides. In a small cross validation experiment, an optimal value for p was determined to be equal to 0.1.

The predictive performance using this ad-hoc modification scheme is shown in Table 1 through Table 3 as SMM-PFR. From the table, it is apparent that the modification improves the predictive performance for all alleles in both data sets. The average performance in terms of AUC for the SMM-align method on the six alleles common to the IEDB and AntiJen data sets is 0.729. Using the proposed modification scheme this number is increased to 0.748. For the alleles in the IEDB data set, the average predictive performance of the SMM-align method is 0.730. This value is increased to 0.749 using the PFR modification scheme. In total the PRF modification scheme improved the predictive performance for 15 of the 17 (14 IEDB and 3 AntiJen) data sets, making the improvement highly statistical significant (p = 0.001, Fishers exact test).

An attempt to directly encode the amino acids composition of the PFR's as input to the SMM-align method gave further improvements to the prediction accuracy. Here, the SMM weight matrix was extended to a length of 11 to incorporate the effect of PFR's. PFR's were encoded to the SMM-align method as the average Blosum62 score [21] over a maximum length of three amino acids. The average predicted performance in terms of the AUC using this PFR encoding scheme in combination with the penalty for longer peptides and short amino terminal peptide flanking residues was 0.756 for the alleles in the IEDB data set, and 0.750 for the six alleles in the combined AntiJen and IEDB data set. The performance excluding PRF sequence encoding and including only the penalty for longer peptides and short amino terminal peptide flanking residues was 0.749, and 0.748, respectively, for the two datasets. The gain in predictive performance is minor. However, the performance is consistently increased for all three alleles in AntiJen data set, and is increased for 11 of the alleles in the IEDB data set, making the improvement highly statistical significant (p = 0.001, Fishers exact test) suggesting that amino acid composition of the PFRs does play some role in stabilizing the peptide:MHC complex.

### Mouse H2-IA alleles

Next, the SMM-align method was applied to derive a method for prediction of MHC-II binding affinities for set of three mouse H2-IA alleles in the benchmark dataset. The predictive performance of the method was estimated using five-fold cross-validation. The methods SMM-align, ARB [12] and PredBalbc [22] were included in the benchmark. Table 4 gives the results in terms of the AUC predictive performance of the benchmark calculation.

**Table 4:** Summary of the mouse H2-IA benchmark.

	**AUC**				
**Allele**	**SMM**	**NetMHCII**	**ARB**	**PredBalbc**	**N**

H-2-IAb	0.913	0.908	0.662		76
H-2-IAd	0.819	0.818	0.819	0.659	342
H-2-IAs	0.877	0.898			126

The predictive performance is shown in terms of the average area under the ROC curve. The methods included in the benchmark are SMM (SMM-align), *NetMHCII *(the extended SMM-align method including direct encoding of peptide flanking residues and penalties for longer peptides and short amino terminal peptide flanking residues), ARB [12], and PredBalbc [22]. The performance of the SMM and *NetMHCII *methods was estimated using five-fold cross-validation as described in Methods.

The table demonstrates the predictive power of the SMM-align method, as the performance is higher than or comparable to that of the ARB method. Note that caution should also here be taking when evaluating the predictive performance of the ARB method. This method has been trained on data from the IEDB database, and thus very likely has been trained on data included in the evaluation set. The PredBalbc method seems to perform significantly worse that the other two methods. Noteworthy is the limited gain in predictive performance of the SMM align method when including peptide flanking residues and penalty for long peptides and short amino terminal peptide flanking residues. Here, the H2-IAb allele shows a drop in predictive performance when including PRFs. However, the H2-IAb allele is trained on very limited amount of peptide data, and one could speculate that PRF might improve the predictive performance also for this allele, as more peptide training data becomes available.

### The final NetMHCII prediction method

The final MHC class II prediction method covers 14 HLA-DR and three H2-IA alleles. For each allele, the method is trained in a five-fold cross-validated manner as described in Methods using multiple sequence encoding schemes, Gibbs sampler derived position specific weight matrices, direct encoding of PRFs and penalties for longer peptides and short amino terminal peptide flanking residues. We denote the final method *NetMHCII.*

### Visualization of the peptide binding motifs

**Figure 2:**
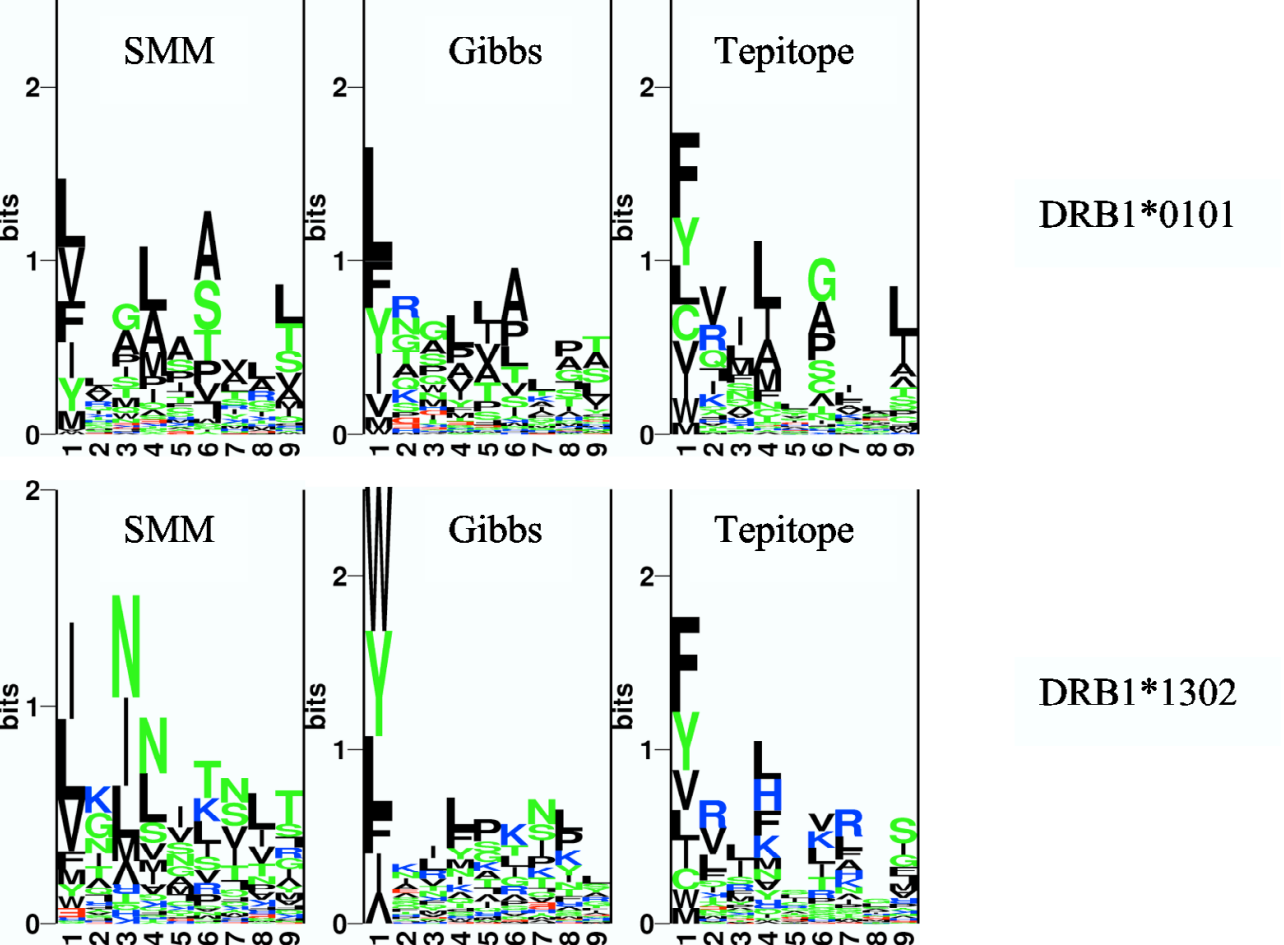
Kullback-Leibler logo visualizations of peptide binding motifs. The upper panel depicts the motif for the DRB1*0101 allele, and the lower panel the motif for the DRB1*1302 alleles. From left the different columns show the motif estimated by the SMM (*NetMHCII*), Gibbs sampler, and TEPITOPE methods, respectively. The height of a column in the logo is proportional to the relative information content in the sequence motif, and the letter height is proportional to the amino acid frequency [23]

The difference in predictive performance between the SMM-align and TEPITOPE method is striking for several alleles. The binding motifs can be visualized in a highly condensed manner using sequence logos [23]. Figure 2 shows such Kullback-Leibler logos [24] for the binding motifs determined by the SMM-align, Gibbs sampler, and TEPITOPE methods, respectively, for the alleles DRB1*0101, and DRB1*1302. The logos are determined from the top 1% of 10.000 random natural peptides selected from the SWISS-PROT database [25]. For the DRB1*0101 allele, the logos show a clear agreement between all three methods, with three major anchors at positions 1, 4 and 6. However, for the DRB1*1302 allele, the logos are in strong disagreement both with regard to the location of and the preferred amino acids at the anchor positions. The TEPITOPE method identifies the position 1 and 4 as anchors, with all anchors except P1 preferring basic amino acids. The SMM-align method, on the other hand, identifies positions 1 and 3 as primary anchors, all with a strong preference for neural or hydrophobic or neutral amino acids.

## Discussion

We have developed an integrated alignment and motif identification algorithm, SMM-align. The method is a hybrid between the SMM method proposed by Peters et al. [14], and the Gibbs sampler method [4]. The method is trained on quantitative MHC:peptide binding data, allowing for a direct prediction of MHC:peptide binding affinities. The peptide data is encoded to the SMM-align method using several sequence schemes including sparse, Blosum and position specific weight matrix encoding. The binding prediction is determined as the ensemble average over the predictions obtained from the different encoding schemes. The search for the optimal SMM-align solution is performed using a Metropolis Monte Carlo (MC) search [26]. To allow for an effective sampling of the potentially large number of local minima in the weight space, an ensemble average of suboptimal MC solution was included in the SMM-align method. Finally for the human HLA-DR alleles, prior knowledge of the preferred amino acids at the P1 position in the binding motif was implemented to guide the MC search. The final method is termed *NetMHCII *and covers 14 HLA-DR and three mouse H2-IA alleles.

The large-scale MHC class II peptide binding benchmark covering 14 HLA-DR and three mouse H2-IA alleles enabled an evaluation of the predictive performance of a set of different publicly available prediction methods including the Gibbs sampler, TEPITOPE, SVRMHC, MHCpred, and ARB methods. For each allele, the peptide binding data were split into five groups each with minimal sequence overlap and thus ideally suited for cross-validated method validation. The benchmark calculation demonstrated that for the HLA-DR alleles the *NetMHCII *method outperforms most of the other methods. Only the ARB method had a comparable performance. However, a direct comparison to the predictive performance of the ARB method is difficult since the ARB method most likely is trained on the data included in the evaluation sets. The MHCpred method was shown to have the poorest performance. A general tendency was observed for small training data set where the TEPITOPE and *NetMHCII *prediction methods achieve comparable predictive performances, underlining the need for large data sets in order to generate accurate MHC class II prediction methods.

Incorporation of peptide length in to MHC class II binding prediction algorithms as suggested by Chang et al. [11] was demonstrated to result in a potential strong over-fitting of the predictive performance. In a cross-validation experiment using affinity data from both the AntiJen and IEDB databases, the two data sets were shown to have highly different peptide length binding profiles, suggesting that the performance gain reported by Chang et al. not necessarily reflects a genuine feature of MHC class II binding predictions, but could arise as a result of over-fitting the method to a length distribution and binding profile of a particular data set. One can speculate why the two data sets have so different affinity-length profiles. A possible reason could be that a large fraction (13%) of the peptides in the AntiJen data have an unnatural amino acid composition (more than 70% alanin for instance). The fraction of unnatural peptides is less than 1% for the IEDB data set. The similarity in profile between the IEDB and SYFEITHI data sets suggests that short peptides with length less than 13–14 amino acids do indeed bind poorly to MHC class II molecules. Both the IEDB and AntiJen affinity profiles show that the likelihood of binding has limited dependence on peptide length for longer peptides. This is in large disagreement with the length profile for natural MHC-II ligands, where the likelihood of observing a peptide of a given length decreases rapidly as the peptide length passes 16 amino acids. However, here it is important to stress the different nature of the three data sets. Both the IEDB and AntiJen data sets contain quantitative data on *in-vitro *binding of peptides to MHC-II molecules. The SYFPEITHI data set, on the other hand, reflects the length of peptides that are naturally presented through the class II antigen presentation pathway. A major event in this pathway is binding to the MHC-II molecule. The difference in the affinity profile and the profile of natural ligands supports the notion put forward by Nelson et al. [27], that antigen processing continues after peptide binding to the MHC class II molecule. First, the longer peptides bind to MHC class II and are next trimmed by exopeptidases before presentation.

The predictive performance of the SMM-align method is relatively poor when compared to the performance values obtained when predicting peptide binding for MHC class I alleles. Here, the predictive performance in terms of the area under the ROC curve tends to fall in the range 0.9–0.95 depending on the allele and number of data points available for the training [28]. There are many possible explanations for the poor predictive performance. Most importantly, the MHC class II binding motif is more degenerate than that of MHC class I. For MHC class I, the anchor positions are highly conserved, often allowing accommodation of only a few different amino acids. As seen from the binding motifs in Figure 2, the situation is quite different for MHC class II. Here, even the most dominant anchor positions allow for a large number of different amino acids. Due to this high degeneracy one might expect a general lower predictive performance. However, there are other issues affecting the predictive performance of the SMM-align (and most other MHC class II binding prediction) method. The SMM-align method takes as a fundamental assumption that the peptide:MHC binding affinity is determined solely from the nine amino acids in binding core motif. This is clearly a large oversimplification since it is known that peptide flanking residues (PFR) on both sides of the binding core may contribute to the binding affinity and stability [29]. An example of such influence of the peptide flanking amino acids can be observed for the DRB1*0401 restricted peptide WIILGLNK*IVRMYSPTS*I. Here, the core region (IVRMYSPTS) as identified by both the SMM-align, and TEPITOPE methods, is highlighted in italic. The binding affinity for the peptide is 1.37 nM. However, also a truncated version of the peptide exists in the data set, LNK*IVRMYSPTS*I. This peptide shares the binding core sequence with the complete peptide, but its binding affinity is 100 fold lower (177.80 nM). This example clearly illustrates the significance of the peptide flanking amino acids in determining the peptide binding affinity. Here, we have incorporated the PFRs by directly encoding the amino acids composition of the PFR's as input to the SMM-align method, and as an ad-hoc strategy that disfavors binding registers with short amino terminal PRF-length and binding of longer peptides, and demonstrated that these PFR modification schemes indeed lead to a significant improvement in predictive performance.

Comparing the binding motifs identified by the *NetMHCII *and TEPITOPE method highlighted a series of fundamental discrepancies. For the DRB1*1302 allele, for instance, the TEPITOPE method favors basic amino acids at most anchor positions, whereas the *NetMHCII *method identifies a preference for hydrophobic or neutral amino acids at the anchors. The TEPITOPE and *NetMHCII *methods are very different in nature. The TEPITOPE binding motif is derived using "virtual" matrices obtained by alignment of binding pocket amino acids and experimentally derived binding specificities [3]. The *NetMHCII *binding motif, on the other hand, is derived directly from peptide binding data. It remains to be determined which amino acids preference conforms to the experimental binding motif.

Other groups have reported prediction algorithms with very high predictive performance values also for MHC class II binding. However, these studies have been limited to small data sets covering a single or a few different MHC molecules [6,8,30]. Here, we have designed a benchmark setup allowing for large-scale validation and comparison of MHC class II prediction algorithms. Future work based on this type of large-scale benchmark analysis should help identifying which methodologies are most suitable for development of algorithms for MHC class II binding.

All peptide data for the 14 HLA-DR and three mouse H2-IA alleles as well as the SMM-align prediction method (*NetMHCII*) are made publicly available [31,32].

## Methods

### Data

Quantitative peptide:HLA binding data were downloaded from the IEDB database November 2006 [13]. Only HLA DR alleles with more than 100 unique peptides and mouse H2-IA data with more than 75 unique peptides were included. The final data set covers 14 HLA-DR and three H2-IA alleles, with a total number of peptide IC50 values of 5147. This dataset is thereafter referred to as the IEDB data set.

The SMM-align method includes a weight matrix encoding the amino acids preferences identified by a Gibbs sampler trained on HLA ligand data (peptides known to bind a given HLA complex). HLA ligand data were downloaded from the SYFPEITHI database [33]. Only peptides of nine amino acids length or more and peptides not present in the IEDB data set were included. A total of 360 HLA ligands were included in the SYF data set.

A summary of the data is shown in Table 5

**Table 5:** Data included in the benchmark calculation.

**HLA-DR alleles**	**IEDB**	**# Binders**	**SYFPEITHI**
DRB1*0101	1203	920	33
DRB1*0301	474	65	22
DRB1*0401	457	209	65
DRB1*0404	168	74	23
DRB1*0405	171	88	23
DRB1*0701	310	125	34
DRB1*0802	174	58	1
DRB1*0901	117	47	13
DRB1*1101	359	95	23
DRB1*1302	179	101	20
DRB1*1501	365	188	12
DRB3*0101	102	3	2
DRB4*0101	181	74	5
DRB5*0101	343	112	11
**H2-IAb**	76	43	47
**H2-IAd**	342	56	7
**H2-IAs**	126	35	19

The first column gives the allele name, the second to fourth columns the number of unique peptide data, and binders included for each allele in the IEDB [13] and SYFPEITHI [33] databases, respectively. Binding peptides were identified using an IC50 binding threshold of 500 nM.

Designing a benchmark is quite more difficult for Class II binding prediction compared to Class I due to the broad length variation between the different peptides, and the potential data redundancy this imposes. To make an evaluation of a prediction method, one has to define the evaluation set so that none of the 9 mer sequences of the evaluation set are present in the training data. We have designed a simple Hobohm1 [34] inspired algorithm that aims at minimizing the overlap between training and evaluation data. The algorithm is applied to all peptide data for each allele. For each allele: Add each new peptide to list of non-redundant peptide sequences, NR, if it has no identical nonamer peptide overlap with any of the peptides already on the NR list. Otherwise the new peptide is added to the cluster defined by the first hit on the NR list. Next all peptides in the NR list (together with their cluster members) are split into five subsets. We are aware that this approach does not ensure zero overlap on the 9 mer level between the different data subsets. However, the overlap is minor and for most alleles in the order 0.5–2% (data not shown).

### Methods

#### Gibbs sampler weight matrices

For each allele, a weight matrix describing the binding motif was constructed based on the relevant data in the SYF data set and the set of binding peptides in the IEDB training set, using the Gibbs sampler method as described by Nielsen et al. [4]. An IC50 value of 500 nM was used identify peptide binders from the IEDB data set.

#### The SMM-align method

The binding motif for all MHC class II alleles is defined in terms of a 9 × 20 weight matrix, where 9 is the length of the binding motif, and 20 the number of different amino acids. The SMM-align method seeks to identify a weight matrix that optimally reproduces the measured IC50 values for each peptide. Inspired by the work on MHC class I binding, the IC50 affinity values in nM units are log-transformed using the relation 1 - log_50k_(IC50 nM), before optimizing the weights in the matrix [35]. Peptides with affinity values greater than 50,000 nM are assigned a log-transformed value of zero. The weight matrix is next optimized so that the mean square error between predicted and measured log_50k_(IC50) values is minimal.

The predicted binding affinity for a peptide sequence is determined as the highest nonamer peptide score within the peptide, where a nonamer peptide score is calculated as

s=∑l∑a'vla'a⋅wla',
 MathType@MTEF@5@5@+=feaafiart1ev1aaatCvAUfKttLearuWrP9MDH5MBPbIqV92AaeXatLxBI9gBaebbnrfifHhDYfgasaacH8akY=wiFfYdH8Gipec8Eeeu0xXdbba9frFj0=OqFfea0dXdd9vqai=hGuQ8kuc9pgc9s8qqaq=dirpe0xb9q8qiLsFr0=vr0=vr0dc8meaabaqaciaacaGaaeqabaqabeGadaaakeaacqWGZbWCcqGH9aqpdaaeqbqaamaaqafabaGaemODay3aa0baaSqaaiabdYgaSjabdggaHjabcEcaNaqaaiabdggaHbaaaeaacqWGHbqycqGGNaWjaeqaniabggHiLdaaleaacqWGSbaBaeqaniabggHiLdGccqGHflY1cqWG3bWDdaWgaaWcbaGaemiBaWMaemyyaeMaei4jaCcabeaakiabcYcaSaaa@45B0@

where w_la' _is the binding motif weight at position ***l ***for amino acid ***a'***, and ***v*^*a*^_*la*'_**, is the sequence-encoding value for amino acid **a' **for amino acid **a**. The peptide sequences are presented to the SMM-align method using several sequence-encoding schemes. The first is the conventional sparse encoding where each amino acid is encoded as a 20-digit binary number (a single 1 and 19 zeros). The second is the Blosum50 encoding in which the amino acids are encoded as the Blosum50 score for replacing the amino acid with each of the 20 amino acids [21]. Note that since the sequence encoding for each amino acid thus is a constant "vector", the relation for the peptide score can be simplified to the conventional scoring scheme

s=∑lula,whereula=∑a'vla'a⋅wla'.
 MathType@MTEF@5@5@+=feaafiart1ev1aaatCvAUfKttLearuWrP9MDH5MBPbIqV92AaeXatLxBI9gBaebbnrfifHhDYfgasaacH8akY=wiFfYdH8Gipec8Eeeu0xXdbba9frFj0=OqFfea0dXdd9vqai=hGuQ8kuc9pgc9s8qqaq=dirpe0xb9q8qiLsFr0=vr0=vr0dc8meaabaqaciaacaGaaeqabaqabeGadaaakeaafaqabeqadaaabaGaem4CamNaeyypa0ZaaabuaeaacqWG1bqDdaWgaaWcbaGaemiBaWMaemyyaegabeaaaeaacqWGSbaBaeqaniabggHiLdGccqGGSaalaeaacqqG3bWDcqqGObaAcqqGLbqzcqqGYbGCcqqGLbqzaeaacqWG1bqDdaWgaaWcbaGaemiBaWMaemyyaegabeaakiabg2da9maaqafabaGaemODay3aa0baaSqaaiabdYgaSjabdggaHjabcEcaNaqaaiabdggaHbaaaeaacqWGHbqycqGGNaWjaeqaniabggHiLdGccqGHflY1cqWG3bWDdaWgaaWcbaGaemiBaWMaemyyaeMaei4jaCcabeaaaaGccqGGUaGlaaa@5721@

For both the sparse and Blosum encoded SMM-align matrices, the prediction scores thus converts to a simple matrix sum.

The final prediction score for a nonomer peptide is calculated as the average of the sparse and Blosum encoded predictions.

A Metropolis Monte Carlo (MC) procedure [26] is invoked to search for the optimal weight matrix. Initially, random weights are assigned to the matrix keeping the sum at each position equal to zero. In each Monte Carlo step, a position is selected at random, and the weight on two amino acids are updated keeping the sum of the weights equal to zero. The energy function guiding the Monte Carlo search is

E=1N∑i(mi−si)2+λ⋅1L⋅A∑l∑awla 2,
 MathType@MTEF@5@5@+=feaafiart1ev1aaatCvAUfKttLearuWrP9MDH5MBPbIqV92AaeXatLxBI9gBamXvP5wqSXMqHnxAJn0BKvguHDwzZbqegyvzYrwyUfgarqqtubsr4rNCHbGeaGqiA8vkIkVAFgIELiFeLkFeLk=iY=Hhbbf9v8qqaqFr0xc9pk0xbba9q8WqFfeaY=biLkVcLq=JHqVepeea0=as0db9vqpepesP0xe9Fve9Fve9GapdbaqaaeGacaGaaiaabeqaamqadiabaaGcbaGaemyrauKaeyypa0ZaaSqaaSqaaiabigdaXaqaaiabd6eaobaakmaaqafabaGaeiikaGIaemyBa02aaSbaaSqaaiabdMgaPbqabaaabaGaemyAaKgabeqdcqGHris5aOGaeyOeI0Iaem4Cam3aaSbaaSqaaiabdMgaPbqabaGccqGGPaqkdaahaaWcbeqaaiabikdaYaaakiabgUcaRGGaciab=T7aSjabgwSixpaaleaaleaacqaIXaqmaeaacqWGmbatcqGHflY1cqWGbbqqaaGcdaaeqbqaamaaqafabaGaem4DaC3aa0baaSqaaiabbYgaSjabbggaHbqaaiabbccaGiabbkdaYaaaaeaacqWGHbqyaeqaniabggHiLdaaleaacqWGSbaBaeqaniabggHiLdGccqGGSaalaaa@66B0@

where *s*_*i *_is the prediction score, *m*_*i *_is the measured (log-transformed) binding affinity, N is the number of peptide data, L is the binding motif length, A is the number of different amino acids, w_la _are the weight matrix elements, and a term weighted by a parameter λ is introduced to avoid over-fitting. This term penalizes high weights and thereby forces weights that do not significantly lower the energy function towards small values. In a small-scale cross-validation experiment using only sparse sequence encoding, the parameter λ was determined to have an optimal value of 0.02.

The SMM-align method can readily be extended to include the Gibbs sampler weight matrix, by expanding the space of the SMM weights to include additional weights for the L positions in the Gibbs sampler matrix. In the Monte Carlo search, the number of weights is then 189 for a nonamer binding motif. Only non-negative values are allowed for the weights on the Gibbs sampler matrix. The final weight matrix is determined as the weighted mean of the SMM-align and Gibbs sampler matrices, with relative weights on the Gibbs sampler matrix determined from the Monte Carlo search.

As demonstrated earlier, the Gibbs sampler performance can be greatly improved by restricting the number of allowed amino acids at the P1 position in the binding motif [4]. We adopt a similar approach, to improve the performance of the SMM-align method for HLA-DR alleles. In the Monte Carlo step modifying the weights at position P1 in the matrix, hydrophobic amino acids (ILVMFYW) are forced to take only non-negative values, and non-hydrophobic amino acids are forced to take only non-positive values.

The probability of accepting a move in the Monte Carlo search is determined by the relation

P=min⁡[1,epx(−dET)],
 MathType@MTEF@5@5@+=feaafiart1ev1aaatCvAUfKttLearuWrP9MDH5MBPbIqV92AaeXatLxBI9gBaebbnrfifHhDYfgasaacH8akY=wiFfYdH8Gipec8Eeeu0xXdbba9frFj0=OqFfea0dXdd9vqai=hGuQ8kuc9pgc9s8qqaq=dirpe0xb9q8qiLsFr0=vr0=vr0dc8meaabaqaciaacaGaaeqabaqabeGadaaakeaacqWGqbaucqGH9aqpcyGGTbqBcqGGPbqAcqGGUbGBdaWadaqaaiabigdaXiabcYcaSiabdwgaLjabdchaWjabdIha4jabcIcaOiabgkHiTmaalaaabaGaemizaqMaemyraueabaGaemivaqfaaiabcMcaPaGaay5waiaaw2faaiabcYcaSaaa@4218@

where dE is the difference in energy between the end and start configurations, and T a scaler.

#### SMM-align training

Each MC search was initiated with random weights. The scalar T was initialized to 0.01 and lowered to 0.000001 in 20 uniform steps. At each value of T, 2500 Monte Carlo moves were performed. The acceptance of a move was determined using Equation 2. The motif length, ***L***, was fixed at nine amino acids.

The configuration-space of the peptide sequences contains many local minima with close to identical energy. In order to achieve an effective sampling of these local minima, the MC calculations were repeated 25 times with different initial weight configurations. For each run the final energy and weight matrix were recorded, and the top 10 scoring matrices were kept in the matrix ensemble.

In the five-fold cross-validated training the peptides were split into five subsets as described earlier. One of the subsets were left out from the SMM training and used as evaluation set. The remaining subsets were used to train the weight matrix. In this manner, all peptides will in turn be part of the evaluation set, and the predictive performance can be estimated on all data. This approach lowers the possible effects of over-fitting while keeping the size of data set for evaluation maximal.

#### SVRMHC predictions

The SVRMHC predictions were obtained using default parameter setting for the SVRMHC webserver [36]. The server returns pIC50 prediction scores for each nonamer within the query peptide, and the maximum score was assigned as the binding pIC50 prediction value for the query peptide.

#### MHCpred predictions

The MHCpred predictions were obtained using default parameter setting for the MHCpred webserver [37]. The server returns IC50 prediction scores for each nonamer within the query peptide, and the minimum score was assigned as the binding IC50 prediction value for the query peptide.

#### ARB predictions

The ARB predictions were obtained using default parameter setting for the ARB webserver [38].

## Authors' contributions

MN developed the SMM-align method, designed the MHC class II benchmark, trained the prediction method and did the performance comparison between the different prediction methods. CL prepared in the IEDB and SYFPEITHI peptide data sets. All authors read and corrected the manuscript.

## Supplementary Material

Additional file 1 Details of the benchmark calculation covering the 14 HLA-DR alleles. The predictive performance is shown in terms of the Pearson's correlation (upper table) and the Spearsman's rank correlation (lower table) for the SMM-align, Gibbs sampler [1], TEPITOPE [2], SVRMHC [3], MHCpred [4], and ARB methods, respectively. The SMM-PRF method refers to the extended SMM-align method including penalties for long peptides and short amino terminal peptide flanking residues, and the NetMHCII method refers to the final extended SMM align method including direct encoding of peptide flanking residues and penalties for longer peptides and short amino terminal peptide flanking residues. The first column gives the allele names as 1*0101 for DRB1*0101 etc The last column gives the number of peptide data included for each allele. For each allele, the performance of the SMM-align, Gibbs sampler, and NetMHCII methods was estimated using five-fold cross-validation as described in the text. Click here for file

Additional file 2 MHC-II binding affinity as a function of peptide length for three MHC-II alleles, DRB1*0101, DRB1*0401, and DRB1*1501. In the left figure results for the DRB1*0101 allele are displayed, and the right figure shows an average over the 3 alleles. For each data set, the mean binding affinity for peptides of a given length is shown as a function of the peptide length. In black is shown the curves for the data in the AntiJen data set [5]. In red is shown the curves for the data in the IEDB data set [15]. The green curves show histograms of the length distribution of natural MHC ligands as downloaded from the SYFPEITHI database [31]. As suggested by Cheng et al., values for peptide lengths where no affinity data are available are set to the mean binding value over the entire data set. All curves are smoothed using a running mean of length three. It is clear from the figure that the AntiJen and IEDB data sets have very distinct mean binding profiles for short peptides (length < 15 amino acids). In this regime of peptide lengths, the IEDB data set, in contrast to the AntiJen data set, seems to follow an affinity profile in agreement with the observed length profile for natural MHC-II ligands. For longer peptides, both the AntiJen and IEDB data sets follow a similar affinity profile that deviate strongly from the length profile of natural MHC-II ligands. Click here for file
