# Supplementary material for: Prediction of MHC class II binding affinity using SMM-align, a novel stabilization matrix alignment method
Source: BMC Bioinformatics. 2007 Jul 4;8:238. doi: 10.1186/1471-2105-8-238 (PMC1939856; doi:10.1186/1471-2105-8-238)
Supplement: Additional file 1 — Details of the benchmark calculation covering the 14 HLA-DR alleles. The predictive performance is shown in terms of the Pearson's correlation (upper table) and the Spearsman's rank correlation (lower table) for the SMM-align, Gibbs sampler [1], TEPITOPE [2], SVRMHC [3], MHCpred [4], and ARB methods, respectively. The SMM-PRF method refers to the extended SMM-align method including penalties for long peptides and short amino terminal peptide flanking residues, and the NetMHCII method refers to the final extended SMM align method including direct encoding of peptide flanking residues and penalties for longer peptides and short amino terminal peptide flanking residues. The first column gives the allele names as 1*0101 for DRB1*0101 etc The last column gives the number of peptide data included for each allele. For each allele, the performance of the SMM-align, Gibbs sampler, and NetMHCII methods was estimated using five-fold cross-validation as described in the text. [file 1471-2105-8-238-S1.pdf]

Details of the benchmark calculation covering the 14 HLA-DR alleles.

Pearson's correlation

Allele	SMM	Gibbs	TEPITOPE	SCRMHC	MHCpred	ARB	SMM-PRF	NetMHCII	N
1*0101	0.423	0.317	0.186	0.130	0.158	0.409	0.449	0.451	1203
1*0301	0.500	0.426	0.052			0.500	0.461	0.463	474
1*0401	0.461	0.476	0.112	0.115	0.193	0.448	0.485	0.489	457
1*0404	0.479	0.418	0.115			0.521	0.492	0.459	168
1*0405	0.399	0.424	0.129	0.150		0.425	0.417	0.436	171
1*0701	0.491	0.359	0.086		0.301	0.417	0.506	0.522	310
1*0802	0.391	0.370	0.089			0.503	0.410	0.442	174
1*0901	0.399	0.380				0.444	0.443	0.454	117
1*1101	0.373	0.383	0.144			0.423	0.384	0.402	359
1*1302	0.568	0.395	0.432			0.750	0.589	0.583	179
1*1501	0.424	0.325	0.155	0.185		0.551	0.453	0.459	365
3*0101	0.198	0.130				0.277	0.240	0.340	102
4*0101	0.447	0.440				0.513	0.433	0.428	181
5*0101	0.332	0.301	0.123	0.207		0.322	0.336	0.338	343

Spearman's rank correlation

Allele	SMM	Gibbs	TEPITOPE	SVRHMM	MHCpred	ARB	SMM-PRF	NetMHCII	N
1*0101	0.392	0.260	0.333	0.213	0.146	0.376	0.417	0.413	1203
1*0301	0.507	0.453	0.227			0.506	0.441	0.466	474
1*0401	0.468	0.482	0.508	0.461	0.176	0.434	0.495	0.499	457
1*0404	0.509	0.433	0.609			0.529	0.529	0.481	168
1*0405	0.401	0.428	0.542	0.409		0.420	0.400	0.417	171
1*0701	0.491	0.353	0.460		0.309	0.410	0.509	0.531	310
1*0802	0.411	0.375	0.472			0.517	0.442	0.461	174
1*0901	0.430	0.398				0.440	0.477	0.487	117
1*1101	0.390	0.385	0.382			0.421	0.406	0.426	359
1*1302	0.578	0.400	0.411			0.763	0.595	0.594	179
1*1501	0.422	0.305	0.453	0.481		0.561	0.453	0.461	365
3*0101	0.252	0.232				0.276	0.302	0.357	102
4*0101	0.420	0.417				0.507	0.412	0.403	181
5*0101	0.356	0.288	0.313	0.322		0.330	0.346	0.347	343

Supplementary Table 1. The predictive performance is shown in terms of the Pearson's correlation (upper table) and the Spearsman's rank correlation (lower table) for the SMM-align, Gibbs sampler [1], TEPITOPE [2], SVRMHC [3], MHCpred [4], and ARB methods, respectively. The SMM-PRF method refers to the extended SMM-align method including penalties for long peptides and short amino terminal peptide flanking residues, and the *NetMHCII* method refers to the final extended SMM align method including direct encoding of peptide flanking residues and penalties for longer peptides and short amino terminal peptide flanking residues. The first column gives the allele names as 1*0101 for DRB1*0101 etc.. The last column gives the number of peptide data included for each allele. For each allele, the performance of the SMM-align, Gibbs sampler, and *NetMHCII* methods was estimated using five-fold cross-validation as described in the text.
