# Supplementary material for: Prediction of MHC class II binding affinity using SMM-align, a novel stabilization matrix alignment method
Source: BMC Bioinformatics. 2007 Jul 4;8:238. doi: 10.1186/1471-2105-8-238 (PMC1939856; doi:10.1186/1471-2105-8-238)
Supplement: Additional file 2 — MHC-II binding affinity as a function of peptide length for three MHC-II alleles, DRB1*0101, DRB1*0401, and DRB1*1501. In the left figure results for the DRB1*0101 allele are displayed, and the right figure shows an average over the 3 alleles. For each data set, the mean binding affinity for peptides of a given length is shown as a function of the peptide length. In black is shown the curves for the data in the AntiJen data set [5]. In red is shown the curves for the data in the IEDB data set [15]. The green curves show histograms of the length distribution of natural MHC ligands as downloaded from the SYFPEITHI database [31]. As suggested by Cheng et al., values for peptide lengths where no affinity data are available are set to the mean binding value over the entire data set. All curves are smoothed using a running mean of length three. It is clear from the figure that the AntiJen and IEDB data sets have very distinct mean binding profiles for short peptides (length < 15 amino acids). In this regime of peptide lengths, the IEDB data set, in contrast to the AntiJen data set, seems to follow an affinity profile in agreement with the observed length profile for natural MHC-II ligands. For longer peptides, both the AntiJen and IEDB data sets follow a similar affinity profile that deviate strongly from the length profile of natural MHC-II ligands. [file 1471-2105-8-238-S2.pdf]

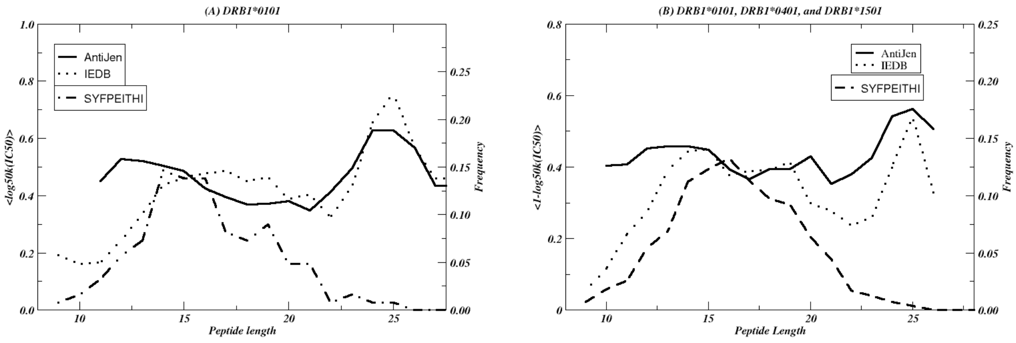

Supplementary Figure 1. MHC-II binding affinity as a function of peptide length for three MHC-II alleles, DRB1*0101, DRB1*0401, and DRB1*1501. In the left figure results for the DRB1*0101 allele are displayed, and the right figure shows an average over the 3 alleles. For each data set, the mean binding affinity for peptides of a given length is shown as a function of the peptide length. In black is shown the curves for the data in the AntiJen data set [5]. In red is shown the curves for the data in the IEDB data set [15]. The green curves show histograms of the length distribution of natural MHC ligands as downloaded from the SYFPEITHI database [31]. As suggested by Cheng et al., values for peptide lengths where no affinity data are available are set to the mean binding value over the entire data set. All curves are smoothed using a running mean of length three. It is clear from the figure that the AntiJen and IEDB data sets have very distinct mean binding profiles for short peptides (length < 15 amino acids). In this regime of peptide lengths, the IEDB data set, in contrast to the AntiJen data set, seems to follow an affinity profile in agreement with the observed length profile for natural MHC-II ligands. For longer peptides, both the AntiJen and IEDB data sets follow a similar affinity profile that deviate strongly from the length profile of natural MHC-II ligands.
